# Moderating effect of resilience on the associations of perceived stress and anxiety with unhealthy alcohol use and insomnia in Chinese hospital staff

**DOI:** 10.3389/fpubh.2026.1779761

**Published:** 2026-05-19

**Authors:** Shijie Xu, Guohai Yang, Xianhua Xu, Mingji Li

**Affiliations:** 1 Medical Research Center, Hainan Cancer Hospital, Affiliated Cancer Hospital of Hainan Medical University, Haikou, Hainan, China; 2 Medical Research Center, Institute of Human Behavioral Medicine, Seoul National University, Seoul, Republic of Korea; 3 Department of Sociology, Hong Kong Shue Yan University, Hong Kong, China; 4 Department of Pathology, Hainan Cancer Hospital, Affiliated Cancer Hospital of Hainan Medical University, Haikou, Hainan, China; 5 Department of Education, College of Education, Ewha Womans University, Seoul, Republic of Korea

**Keywords:** perceived stress, anxiety, unhealthy alcohol use, insomnia, resilience, Chinese hospital staff, moderation

## Abstract

**Objective:**

This study examined the relationships among perceived stress, anxiety, unhealthy alcohol use, and insomnia among hospital staff and explored whether resilience moderates these associations.

**Methods:**

We conducted a cross-sectional survey among 508 hospital staff members at a tertiary hospital in Hainan Province, China. Standardized psychometric instruments were employed, including the Perceived Stress Scale (PSS-10), Generalized Anxiety Disorder Scale (GAD-7), Connor–Davidson Resilience Scale (CD-RISC-10), Alcohol Use Disorder Identification Test (AUDIT), and Insomnia Severity Index (ISI). Statistical analyses included descriptive statistics, *t*-tests/ANOVA, Pearson correlations, and moderation analyses using the PROCESS Macro (Model 1) with bootstrapping (5,000 samples, 95% CI).

**Results:**

Independent samples *t*-tests and ANOVA revealed that perceived stress, anxiety, resilience, and unhealthy alcohol use differed significantly across occupational, socioeconomic, and personal characteristics. Correlation analysis indicated that perceived stress and anxiety were positively correlated with unhealthy alcohol use and insomnia, while resilience was negatively associated with all adverse outcomes. PROCESS moderation analysis demonstrated that resilience significantly moderated the relationships between perceived stress/anxiety and unhealthy alcohol use; these associations were significant at low and moderate resilience levels but non-significant at high resilience levels. For insomnia, the associations remained significant across all resilience levels but progressively weakened as resilience increased.

**Conclusion:**

Resilience moderates the associations between perceived stress/anxiety and unhealthy alcohol use/insomnia, with higher resilience levels progressively attenuating these associations. These findings highlight the importance of implementing systematic resilience-building interventions to safeguard the mental and behavioral health of healthcare workers.

## 1 Introduction

Healthcare workers experience substantially higher stress levels than individuals employed in other occupations; in particular, they exhibit elevated rates of depression and anxiety compared with those in the general workforce (1, 2). Healthcare workers face exceptionally demanding work environments marked by extended shifts, the management of critically ill patients, and an ongoing emotional burden (3). They frequently encounter workplace hazards, such as burnout, violence, and heavy workloads (4, 5). Recent research indicates that healthcare workers' anxiety and depression symptoms are significantly associated with work stress and may impair their work capacity (6). These mental health conditions will continue to escalate unless a thorough investigation is made of their development and progression among healthcare personnel, and the consequences will extend far beyond individual suffering. Therefore, understanding the healthcare-specific psychological sequelae and their ripple effects is essential for developing targeted interventions that effectively protect healthcare workers from occupational mental health deterioration (7).

Sleep disturbances, particularly insomnia, represent a prominent manifestation of occupational stress among healthcare workers (8, 9). Indeed, the implications of sleep disturbance extend across multiple domains of an individual's health and occupational performance. For example, acute insomnia impairs critical cognitive functions such as reaction time, memory consolidation, and executive decision-making, directly threatening patient safety (10). Similarly, chronic insomnia precipitates severe long-term health consequences, including major depressive disorder, cardiovascular disease, and burnout syndrome (11, 12). Studies have demonstrated that insomnia among healthcare workers reduces clinical performance (13), increases absenteeism, and reduces overall productivity (14). Epidemiological studies have reported that the prevalence of sleep disorders among healthcare professionals ranges from 38.6 to 69.7%, which significantly exceeds the rate of sleep disorders in the general population (15–17). This study examines whether hospital staff members suffering from occupational stress and anxiety have an increased risk of sleep disturbances in healthcare settings.

Hospital staff members who are experiencing occupational stress and anxiety are more likely to regularly drink alcohol or binge drink. Research has demonstrated that medical students with alcohol dependence show significant correlations with depression (18), while elevated levels of perceived stress and anxiety are associated with substantially higher risks of alcohol misuse and alcohol use disorder (19). In a cross-sectional UK study, doctors who coped with stress through substance use had dramatically increased odds of alcohol dependence (OR = 6.165), binge-drinking (OR = 6.355), and frequent alcohol consumption (OR = 18.836) (20). Another study demonstrated that night-shift healthcare workers often resort to alcohol use or sedatives for sleep and stress reduction, but such self-medication endangers the wellbeing of the staff and threatens patient safety (21). Some healthcare workers suffering from high occupational stress may utilize alcohol to cope with this additional trauma and pressure (22). Although alcohol use may provide temporary relief, it fails to reduce stress over time and actually worsens psychological distress, creating a harmful cycle (23). In light of these findings, we hypothesized that the relationship between perceived stress/anxiety and unhealthy alcohol use would be significantly higher among hospital staff in high-stress healthcare settings.

Although perceived stress or anxiety may increase the likelihood of unhealthy alcohol use or insomnia, not all individuals with high levels of perceived stress or anxiety develop these adverse outcomes, which suggest that resilience may play a moderating role. Indeed, the results of a previous study indicate that resilience buffers the negative effect of stress on insomnia, thereby reducing the risk of impaired sleep quality (24). Similarly, research has demonstrated that resilience functions as a protective factor against problematic drinking behaviors, even under stressful conditions (25). One study found that resilience was negatively correlated with stress and anxiety and positively associated with enhanced psychological adjustment (26). Thus, these findings suggest that the strength of the effects of perceived stress and anxiety on unhealthy alcohol use and insomnia depends on an individual's level of resilience. Individuals with higher levels of resilience are more likely to maintain psychological stability, avoid maladaptive coping strategies such as excessive alcohol use, and experience fewer sleep disturbances as a result. Conversely, individuals with low resilience may be more vulnerable to maladaptive responses when faced with occupational stress and anxiety, potentially leading to unhealthy alcohol use and insomnia. Accordingly, we hypothesized that resilience moderates the relationship between perceived stress/anxiety and unhealthy alcohol use/insomnia among hospital staff.

**Figure 1:**
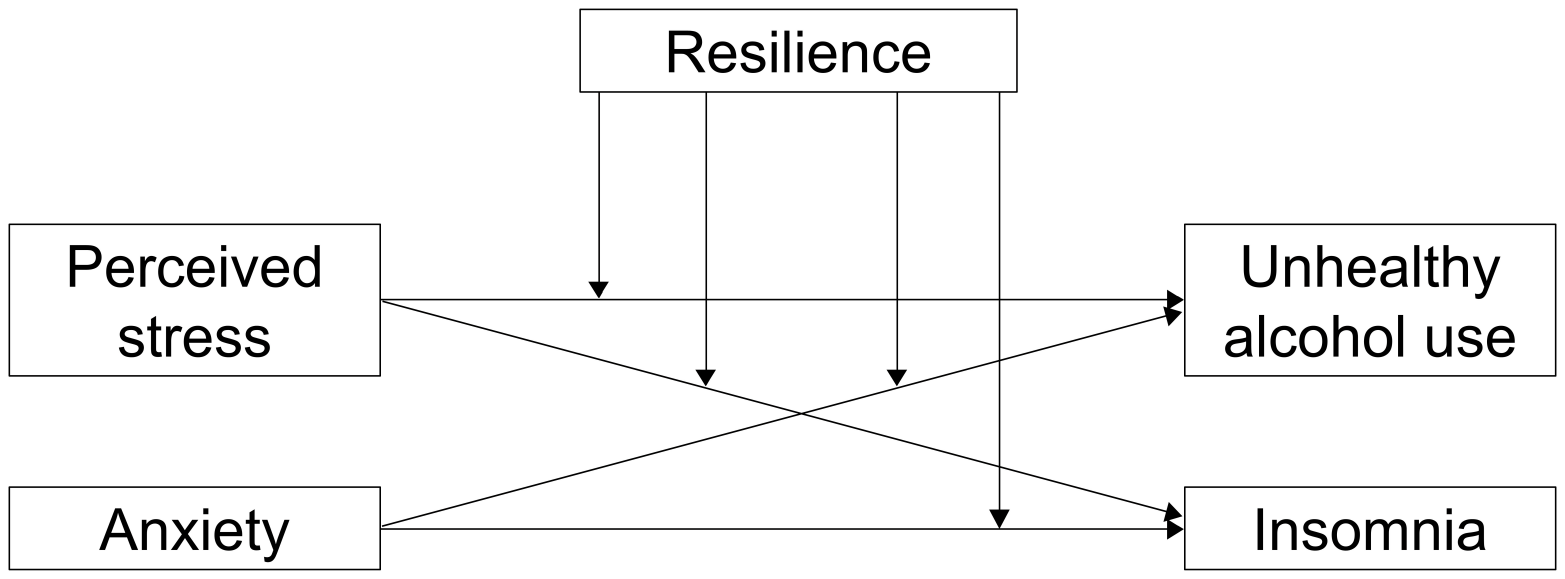
The proposed conceptual framework for the hypothetical model.

Based on this theoretical framework and prior empirical evidence, this study explores the relationships among perceived stress, anxiety, unhealthy alcohol use, and insomnia in Chinese hospital staff. In addition, it investigates whether resilience moderates the associations between perceived stress/anxiety and unhealthy alcohol use/insomnia. Identifying such protective mechanisms can inform targeted interventions to strengthen resilience and safeguard mental health among hospital staff. The conceptual framework of the present study is illustrated in Figure 1, and the following hypotheses were proposed:

H1: Perceived stress, anxiety, resilience, unhealthy alcohol use, and insomnia differ significantly across demographic characteristics among hospital staff.
H2a: Perceived stress is positively correlated with unhealthy alcohol use among hospital staff.
H2b: Perceived stress is positively correlated with insomnia among hospital staff.
H2c: Anxiety is positively correlated with unhealthy alcohol use among hospital staff.
H2d: Anxiety is positively correlated with insomnia among hospital staff.
H3a: Resilience moderates the association between perceived stress and unhealthy alcohol use among hospital staff.
H3b: Resilience moderates the association between perceived stress and insomnia use among hospital staff.
H3c: Resilience moderates the association between anxiety and unhealthy alcohol use among hospital staff.
H3d: Resilience moderates the association between anxiety and insomnia among hospital staff.

## 2 Materials and methods

### 2.1 Study design and participants

A cross-sectional design was adopted to examine the moderating effect of resilience on the relationships among perceived stress, anxiety, unhealthy alcohol use, and insomnia. This approach provides a practical method for capturing the psychological states and behavioral patterns of hospital staff at a defined point in time, and is consistent with the exploratory nature of this investigation. The target population comprised hospital staff, including doctors, nurses, and non-medical personnel, at a tertiary hospital in Hainan Province of China. The data were collected using a standardized paper-based questionnaire administered between March and May 2025. This study was approved by the Institutional Research Ethics Committee of the Affiliated Cancer Hospital of Hainan Medical University.

The inclusion criteria were as follows: (1) current employment at Hainan Cancer Hospital, (2) voluntary participation, and (3) the provision of written informed consent after receiving a complete explanation of the study. All of the participants' responses to the questionnaires were anonymous. Of the 600 individuals who received the survey, 545 (90.83%) returned completed questionnaires. Following data screening, 37 questionnaires containing missing data or logical inconsistencies were excluded, yielding a final sample of 508 valid responses for analysis.

### 2.2 Measurements tools

#### 2.2.1 Perceived stress scale (PSS-10)

The PSS-10 is a 10-item self-administered psychometric instrument designed to assess an individual's perception of stress (27). Each item is scored on a 5-point Likert scale ranging from 0 (never) to 4 (very often), with a total score ranging from 0 to 40. Specifically, six of the items (labeled 1, 2, 3, 6, 9, and 10) are forward-scored, while four of the items (4, 5, 7, and 8) are reverse-scored, with higher values indicating greater stress. The Chinese version of the PSS-10 has demonstrated good internal consistency (Cronbach's α = 0.86) (28). In the present study, the PSS-10 demonstrated satisfactory internal consistency (Cronbach's α = 0.839).

#### 2.2.2 Generalized anxiety disorder scale (GAD-7)

The GAD-7 is a 7-item self-reported psychometric scale that measures an individual's anxiety level (29). Items are rated on a 4-point Likert scale ranging from 0 to 3, yielding total scores from 0 to 21, with higher scores indicating more severe anxiety. The Chinese version of the GAD-7 has demonstrated acceptable reliability and validity (Cronbach's α = 0.898) (30). In the present study, the GAD-7 demonstrated excellent internal consistency (Cronbach's α = 0.929).

#### 2.2.3 Connor-davidson resilience scale (CD-RISC-10)

The CD-RISC-10 is a 10-item self-administered psychometric scale that assesses an individual's mental resilience (31). The items are rated on a 5-point Likert scale ranging from 0 to 4, producing total scores from 0 to 40, with higher scores reflecting greater resilience. The Chinese version of the CD-RISC-10 has demonstrated good internal consistency (Cronbach's α = 0.91) (32). In this study, internal consistency was excellent (Cronbach's α = 0.957).

#### 2.2.4 Alcohol use disorder identification test (AUDIT)

The AUDIT was designed by the World Health Organization to identify an individual's unhealthy alcohol use (33). This 10-item instrument includes five response options (scored 0 to 4 points) for items 1–8 and three response options (scored 0, 2, or 4 points) for items 9–10. Total scores range from 0 to 40, with higher scores indicating a greater risk for harmful alcohol use and potential alcohol dependence. The Chinese version of the AUDIT has demonstrated adequate validity and reliability (Cronbach's α = 0.714) (34). In the present study, the AUDIT demonstrated good internal consistency (Cronbach's α = 0.855).

#### 2.2.5 Insomnia severity index (ISI)

The ISI is a 7-item self-reported scale that assesses the severity of insomnia symptoms (35). The items are rated on a 5-point Likert scale ranging from 0 to 4, with total scores ranging from 0 to 28; higher scores indicate more severe insomnia. The Chinese version of the ISI has demonstrated adequate psychometric properties (Cronbach's α = 0.83) (36). In the present study, the ISI demonstrated excellent internal consistency (Cronbach's α = 0.913).

#### 2.2.6 Covariates

The following demographic and socioeconomic characteristics were included as covariates in the analyses: sex (male, female), age (< 25, 25–35, 36–45, >45 years), marital status (unmarried, married, divorced/widowed), occupation (doctor, nurse, non-medical staff), educational level (high school, bachelor's degree, master's degree, doctoral degree), years of work experience (< 2, 2–5, 6–10, 11–15, >15), weekly working hours (< 40, 40–48, >48), and monthly income [ < 5,000, 5,000–10,000, 10,000–20,000, >20,000 CNY (Chinese Yuan)].

### 2.3 Statistical analyses

All of the statistical analyses were performed using SPSS version 22.0 (IBM Corp., Armonk, NY, USA). Descriptive statistics, including means, standard deviations (SDs), frequencies, and percentages, were calculated to characterize the sample. Independent samples *t*-tests were used for comparisons between two groups (i.e., gender), while one-way ANOVA tests were used for comparisons among more than two groups. In cases where the ANOVA revealed significant differences, Scheffé's *post-hoc* test was applied to determine the source of the difference. A Pearson's correlation analysis was used to examine bivariate relationships among the study variables. The moderating effect of resilience on the associations between perceived stress/anxiety and unhealthy alcohol use/insomnia was examined using Model 1 of the PROCESS Macro for SPSS, version 4.3 (37). To further explore the moderation effects, simple slopes analyses were conducted to examine the conditional effects of perceived stress and anxiety on unhealthy alcohol use and insomnia at three levels of resilience: low (1 SD below the mean), mean, and high (1 SD above the mean). Gender, age, marital status, occupation, educational level, years of work experience, weekly working hours, and income were included as covariates in the moderation analyses. The significance of conditional effects was evaluated using bootstrapping with 5,000 resamples and 95% bias-corrected confidence intervals (CIs). A significant effect was indicated when the 95% CI did not contain a value of zero (38). To reduce multicollinearity, all variables were mean-centered before creating interaction terms. Statistical significance was set at *p* < 0.05.

## 3 Results

### 3.1 Descriptive statistics

The sample was predominantly female, with women comprising approximately two-thirds of the participants (68.31%). The majority of participants were aged 25–35 years (67.72%), and over half were married (54.13%). Nurses represented the largest occupational group (44.29%), followed by doctors (32.28%) and non-medical staff (23.43%). Most participants held a bachelor's degree (71.85%), had 2–10 years of work experience (66.93%), worked 40–48 h per week (61.42%), and earned a monthly income of 5,000–10,000 CNY (50.98%). Detailed distributions for all demographic variables are presented in Table 1.

**Table 1:** Demographics characteristics and the distribution of perceived stress, anxiety, resilience, unhealthy alcohol use, and insomnia (*N* = 508).

Variable	Respondents	Perceived stress		Anxiety		Resilience		Unhealthy alcohol use		Insomnia	
	*N* (%)	Mean ±SD	*P*-value	Mean ±SD	*P*-value	Mean ±SD	*P*-value	Mean ±SD	*P*-value	Mean ±SD	*P*-value
Gender											
Men	161 (31.69%)	18.88 ± 4.613	0.352	6.98 ± 5.234	0.264	25.09 ± 7.588	0.668	7.43 ± 6.264	< 0.001^***^	8.71 ± 5.817	0.620
Women	347 (68.31%)	18.46 ± 4.844		6.40 ± 5.417		24.77 ± 7.966		4.22 ± 5.443		8.44 ± 5.858	
Age (years)											
< 25	57 (11.22%)	17.67 ± 6.160	0.029^*^	5.91 ± 5.546	0.172	25.02 ± 10.054	0.014^*^	4.93 ± 6.138	0.177	8.37 ± 6.326	0.391
25 – 35	344 (67.72%)	19.01 ± 4.545		6.69 ± 5.440		24.49 ± 7.441		5.10 ± 5.839		8.55 ± 5.882	
36 – 45	81 (15.94%)	18.01 ± 4.143		7.17 ± 4.839		24.89 ± 7.853		6.44 ± 5.939		9.07 ± 5.796	
> 45	26 (5.12%)	16.96 ± 5.503		4.77 ± 5.218		29.65 ± 6.151		3.96 ± 5.889		6.81 ± 4.000	
Marital status											
Unmarried	193 (37.99%)	18.86 ± 5.124	0.596	6.45 ± 5.569	0.631	24.31 ± 7.891	0.126	5.13 ± 5.875	< 0.001^***^	8.95 ± 5.940	0.339
Married	275 (54.13%)	18.40 ± 4.518		6.77 ± 5.282		25.49 ± 7.643		4.54 ± 5.391		8.17 ± 5.834	
Divorced or widowed	40 (7.87%)	18.60 ± 4.771		6.00 ± 4.925		23.38 ± 8.714		10.55 ± 6.812		8.90 ± 5.368	
Occupation											
Doctor	164 (32.28%)	18.91 ± 5.117	0.030^*^	6.59 ± 5.386	0.033^*^	25.77 ± 7.282	0.105	4.81 ± 5.767	0.197	8.57 ± 5.743	0.125
Nurse	225 (44.29%)	18.90 ± 4.394		7.13 ± 5.636		24.09 ± 8.404		5.12 ± 6.072		8.97 ± 6.078	
Non-medical staff	119 (23.43%)	17.58 ± 4.865		5.55 ± 4.634		25.11 ± 7.386		6.06 ± 5.726		7.62 ± 5.449	
Educational level											
High school	63 (12.40%)	18.22 ± 4.144	0.580	6.73 ± 4.962	0.974	23.19 ± 7.479	0.308	8.54 ± 6.601	< 0.001^***^	8.59 ± 5.662	0.861
Bachelor's degree	365 (71.85%)	18.60 ± 4.828		6.52 ± 5.512		25.03 ± 8.211		4.83 ± 5.695		8.63 ± 6.030	
Master's degree	53 (10.43%)	19.30 ± 4.241		6.79 ± 4.725		25.32 ± 5.986		4.49 ± 5.669		8.08 ± 5.064	
Doctor's degree	27 (5.31%)	18.00 ± 6.251		6.78 ± 5.618		25.81 ± 6.451		4.56 ± 5.161		7.89 ± 5.243	
Years of work experience											
< 2	46 (9.06%)	17.89 ± 6.983	0.121	4.96 ± 5.404	0.013^*^	25.54 ± 8.823	0.001^**^	3.78 ± 4.747	0.007^**^	6.70 ± 5.304	0.155
2 – 5	178 (35.04%)	19.08 ± 4.196		7.54 ± 5.358		23.53 ± 7.572		6.50 ± 6.060		9.05 ± 6.059	
6 – 10	162 (31.89%)	18.86 ± 4.724		6.57 ± 5.570		25.24 ± 7.815		4.93 ± 6.300		8.74 ± 6.047	
11 – 15	75 (14.76%)	18.01 ± 4.203		5.92 ± 4.693		24.28 ± 7.601		4.47 ± 5.105		8.28 ± 5.306	
> 15	47 (9.25%)	17.43 ± 5.038		5.64 ± 5.084		28.98 ± 6.974		4.19 ± 5.902		8.00 ± 5.389	
Weekly working hours											
< 40	64 (12.60%)	18.63 ± 4.377	0.019^*^	5.20 ± 5.538	0.006^**^	25.17 ± 8.671	0.767	5.05 ± 5.731	0.935	7.80 ± 5.655	0.065
40 – 48	312 (61.42%)	18.17 ± 4.732		6.40 ± 5.224		24.67 ± 7.795		5.22 ± 5.946		8.26 ± 5.890	
> 48	132 (25.98%)	19.57 ± 4.937		7.70 ± 5.425		25.20 ± 7.572		5.37 ± 5.922		9.52 ± 5.734	
Income											
< 5,000	159 (31.30%)	18.70 ± 4.257	0.102	6.80 ± 5.333	0.323	23.78 ± 7.917	0.002^**^	6.42 ± 6.024	0.012^*^	8.52 ± 5.723	0.613
5,000 – 10,000	259 (50.98%)	18.71 ± 5.257		6.78 ± 5.654		24.80 ± 8.096		4.49 ± 5.699		8.76 ± 6.044	
10,000 – 20,000	73 (14.37%)	18.60 ± 3.589		5.53 ± 4.279		26.15 ± 6.317		5.16 ± 5.482		8.04 ± 5.549	
> 20,000	17 (3.35%)	15.76 ± 5.495		6.18 ± 5.065		30.82 ± 6.002		6.00 ± 7.929		7.18 ± 5.114	

^*^p < 0.05, ^**^p < 0.01, ^***^p < 0.001.

Independent samples *t*-tests and one-way ANOVA analyses were conducted to examine differences across various demographic variables. Scheffé's *post-hoc* tests were performed when significant omnibus effects were detected. For perceived stress, participants working more than 48 h per week scored higher than those working 40–48 h (*p* = 0.019). For anxiety, staff working more than 48 h per week scored higher than those working fewer than 40 hours (*p* = 0.009), and nurses reported higher anxiety than non-medical staff (*p* = 0.033). For resilience, participants over 45 years old scored higher than those aged 25–35 (*p* = 0.014), staff with more than 15 years of experience scored higher than those with 2–5 years (*p* = 0.001) and 11–15 years (*p* = 0.031), and those earning more than 20,000 CNY scored higher than those earning less than 5,000 CNY (*p* = 0.006) and 5,000–10,000 CNY (*p* = 0.023). For unhealthy alcohol use, men scored higher than women (*p* < 0.001), and divorced or widowed staff scored higher than both unmarried (*p* < 0.001) and married participants (*p* < 0.001). Participants with a high school education also reported higher unhealthy alcohol use than those with a bachelor's (*p* < 0.001), master's (*p* = 0.003), or doctoral degree (*p* = 0.031), and staff earning less than 5,000 CNY scored higher than those earning 5,000–10,000 CNY (*p* = 0.014). No significant demographic differences were observed in insomnia scores.

### 3.2 Correlations among study variables

As illustrated in Table 2, perceived stress was positively correlated with anxiety (*r* = 0.442, *p* < 0.001), unhealthy alcohol use (*r* = 0.220, *p* < 0.001), and insomnia (*r* = 0.371, *p* < 0.001). Anxiety was also positively correlated with both unhealthy alcohol use (*r* = 0.282, *p* < 0.001) and insomnia (*r* = 0.382, *p* < 0.001). In addition, unhealthy alcohol use was positively correlated with insomnia (*r* = 0.340, *p* < 0.001). Resilience was negatively correlated with all of the study variables: perceived stress (*r* = −0.405, *p* < 0.001), anxiety (*r* = −0.241, *p* < 0.001), unhealthy alcohol use (*r* = −0.431, *p* < 0.001), and insomnia (*r* = −0.423, *p* < 0.001).

**Table 2:** The correlations between the main study variables.

Variable	1	2	3	4	5
1. Perceived stress	1				
2. Anxiety	0.442^***^	1			
3. Resilience	−0.405^***^	−0.241^***^	1		
4. Unhealthy alcohol use	0.220^***^	0.282^***^	−0.431^***^	1	
5. Insomnia	0.371^***^	0.382^***^	−0.423^***^	0.340^***^	1

^***^p < 0.001.

### 3.3 Moderating analyses

The moderating role of resilience was examined using Model 1 of the PROCESS Macro. As shown in Table 3, perceived stress and anxiety served as the independent variables, resilience as the moderator, and unhealthy alcohol use as the dependent variable. Perceived stress (*B* = 0.122, *p* = 0.023), resilience (*B* = −0.297, *p* < 0.001), and the perceived stress × resilience interaction (*B* = −0.019, *p* < 0.001) were significantly associated with unhealthy alcohol use. Similarly, anxiety (*B* = 0.203, *p* < 0.001), resilience (*B* = −0.279, *p* < 0.001), and the anxiety × resilience interaction (*B* = −0.018, *p* < 0.001) demonstrated significant associations with unhealthy alcohol use, indicating the moderating effects of resilience.

**Table 3:** Moderating role of resilience in the association between perceived stress/anxiety and unhealthy alcohol use.

Predictors	B	SE	t	LLCI	ULCI
Perceived stress (independent variable)					
Constant	9.797	1.901	5.153^***^	6.062	13.533
Perceived stress	0.122	0.053	2.277^*^	0.017	0.227
Resilience	−0.297	0.031	−9.547^***^	−0.358	−0.236
Perceived stress × resilience	−0.019	0.005	−3.694^***^	−0.030	−0.009
*R^2^*	0.313				
Δ*R^2^*	0.019				
*F*	13.646^***^				
Anxiety (independent variable)					
Constant	9.456	1.864	5.074^***^	5.794	13.118
Anxiety	0.203	0.042	4.796^***^	0.120	0.285
Resilience	−0.279	0.029	−9.710^***^	−0.335	−0.223
Anxiety × Resilience	−0.018	0.005	−3.890^***^	−0.027	−0.009
*R^2^*	0.341				
Δ*R^2^*	0.020				
*F*	15.133^***^				

^*^p < 0.05, ^***^p < 0.001.

The same analytical approach was applied with using insomnia as the dependent variable. As illustrated in Table 4, perceived stress (*B* = 0.311, *p* < 0.001), resilience (*B* = −0.247, *p* < 0.001), and the perceived stress × resilience interaction (*B* = −0.012, *p* = 0.028) significantly predicted insomnia. Likewise, anxiety (*B* = 0.324, *p* < 0.001), resilience (*B* = −0.259, *p* < 0.001), and the anxiety × resilience interaction (*B* = −0.020, *p* < 0.001) were significant predictors of insomnia. These findings indicate that lower resilience strengthens the association between perceived stress/anxiety and unhealthy alcohol use/insomnia.

**Table 4:** Moderating role of resilience in the association between perceived stress/anxiety and insomnia.

Predictors	B	SE	t	LLCI	ULCI
Perceived stress (independent variable)					
Constant	9.534	1.964	4.854^***^	5.675	13.394
Perceived stress	0.311	0.055	5.637^***^	0.203	0.420
Resilience	−0.247	0.032	−7.697^***^	−0.310	−0.184
Perceived stress × Resilience	−0.012	0.005	−2.202^*^	−0.023	−0.001
*R^2^*	0.252				
Δ*R^2^*	0.007				
*F*	4.851^*^				
Anxiety (independent variable)					
Constant	9.521	1.895	5.025^***^	5.799	13.244
Anxiety	0.324	0.043	7.538^***^	0.239	0.408
Resilience	−0.259	0.029	−8.855^***^	−0.316	−0.201
Anxiety × Resilience	−0.020	0.005	−4.268^***^	−0.029	−0.011
*R^2^*	0.304				
Δ*R^2^*	0.026				
*F*	18.219^***^				

^*^p < 0.05, ^***^p < 0.001.

**Figure 2:**
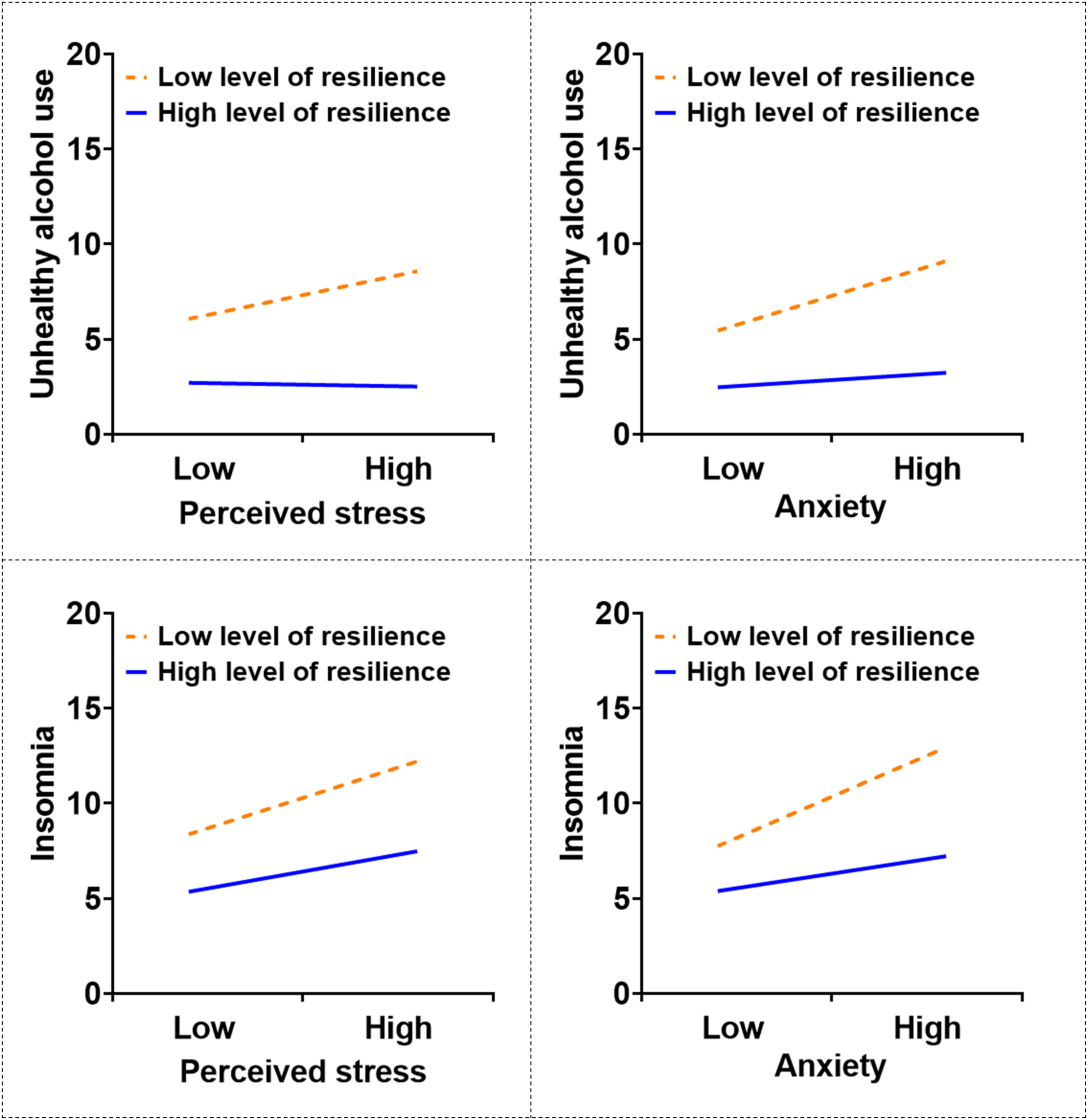
The moderating role of resilience in the association between perceived stress/anxiety and unhealthy alcohol use/insomnia.

The simple slopes analysis (Table 5 and Figure 2) revealed that the association between perceived stress and unhealthy alcohol use was significant at low [*B* = 0.273, *SE* = 0.076, 95% CI (0.123, 0.423)] and moderate resilience levels [*B* = 0.122, *SE* = 0.053, 95% CI (0.017, 0.227)] but non-significant among individuals with high resilience [*B* = −0.030, *SE* = 0.057, 95% CI (−0.142, 0.082)]. A similar pattern emerged for anxiety: Its association with unhealthy alcohol use was significant at low [*B* = 0.343, *SE* = 0.057, 95% CI (0.231, 0.454)] and moderate resilience levels [*B* = 0.203, *SE* = 0.042, 95% CI (0.120, 0.285)] but non-significant at high resilience level [*B* = 0.062, *SE* = 0.054, 95% CI (−0.044, 0.169)].

**Table 5:** Conditional effect results.

Levels of resilience	Unhealthy alcohol use					Insomnia				
	Effect	SE	t	LLCI	ULCI	Effect	SE	t	LLCI	ULCI
Perceived stress (Independent variable)										
Low resilience (M - 1SD)	0.273	0.076	3.576^***^	0.123	0.423	0.405	0.079	5.126^***^	0.250	0.560
Mean resilience (M)	0.122	0.053	2.277^*^	0.017	0.227	0.311	0.055	5.637^***^	0.203	0.420
High resilience (M + 1SD)	−0.030	0.057	−0.523	−0.142	0.082	0.218	0.059	3.706^***^	0.102	0.334
Anxiety (Independent variable)										
Low resilience (M - 1SD)	0.343	0.057	6.041^***^	0.231	0.454	0.480	0.058	8.321^***^	0.367	0.593
Mean resilience (M)	0.203	0.042	4.796^***^	0.120	0.285	0.324	0.043	7.538^***^	0.239	0.408
High resilience (M + 1SD)	0.062	0.054	1.147	−0.044	0.169	0.167	0.055	3.028^**^	0.059	0.275

^*^p < 0.05, ^**^p < 0.01, ^***^p < 0.001.

Regarding insomnia, the associations remained significant across all resilience levels, but they diminished in magnitude as the resilience levels increased. The association between perceived stress and insomnia was significant at low [*B* = 0.405, *SE* = 0.079, 95% CI (0.250, 0.560)], moderate [*B* = 0.311, *SE* = 0.055, 95% CI (0.203, 0.420)], and high [*B* = 0.218, *SE* = 0.059, 95% CI (0.102, 0.334)] resilience levels. Likewise, the association between anxiety and insomnia was significant at low [*B* = 0.480, *SE* = 0.058, 95% CI (0.367, 0.593)], moderate [*B* = 0.324, *SE* = 0.043, 95% CI (0.239, 0.408)], and high [*B* = 0.167, *SE* = 0.055, 95% CI (0.059, 0.275)] resilience levels. Overall, individuals with lower resilience exhibited stronger positive associations between perceived stress/anxiety and unhealthy alcohol use/insomnia.

## 4 Discussion

### 4.1 Demographic differences in perceived stress, anxiety, resilience, unhealthy alcohol use, and insomnia among hospital staff (H1)

Several demographic characteristics revealed meaningful differences in perceived stress, anxiety, resilience, and unhealthy alcohol use among hospital staff. Regarding perceived stress and anxiety, staff working more than 48 h per week reported elevated levels of both measures, and nurses exhibited higher anxiety than non-medical staff, corroborating evidence that direct patient-care roles and extended work schedules impose a greater psychological burden (39, 40). For resilience, higher scores were observed among older staff and those with more years of work experience, suggesting that accumulated occupational experience may foster adaptive coping strategies (41, 42). Higher-income employees also demonstrated greater resilience, consistent with previous findings linking income level to resilience among healthcare workers (43). For unhealthy alcohol use, men scored higher than women, reflecting well-documented gender patterns in drinking behavior (44). Divorced or widowed staff reported higher alcohol consumption, possibly reflecting a reliance on alcohol as a coping mechanism in response to marital loss (45). Lower educational attainment was also associated with elevated alcohol use, which has been attributed to reduced health literacy and limited emphasis on healthy lifestyle behaviors (46). Although insomnia scores did not differ significantly across demographic subgroups, a marginal trend was noted for weekly working hours (*F* = 2.743, *p* = 0.065), with staff working more than 48 h reporting higher scores. This same subgroup also reported significantly elevated perceived stress and anxiety, suggesting that excessive working hours may constitute a common risk factor for both psychological distress and sleep disturbances among hospital staff (47). Collectively, these findings highlight the need for targeted interventions for high-risk subgroups, particularly staff with extended working hours, frontline clinical personnel, and those facing socioeconomic or personal adversity.

### 4.2 The correlation among perceived stress, anxiety, resilience, unhealthy alcohol use and insomnia in hospital staff (H2)

Consistent with H2, perceived stress and anxiety were positively associated with unhealthy alcohol use. This finding aligns with relevant evidence indicating that psychological distress frequently precipitates maladaptive coping mechanisms, particularly alcohol consumption, which individuals often rationalize by citing its perceived anxiolytic properties (48, 49). Perceived stress has also been correlated with problematic drinking patterns (50). Among healthcare professionals, alcohol use disorders intertwine with workplace stressors, simultaneously compromising mental health and diminishing occupational performance (51). Therefore, the observed association between perceived stress/anxiety and unhealthy alcohol use constitute a pathway that warrants targeted intervention strategies. However, this relationship appears bidirectional rather than unidirectional. Different drinking patterns correlate with varying severities of anxiety disorders and psychological strain (52), while alcohol misuse itself exacerbates anxiety, stress, and depression, creating a self-perpetuating cycle of psychological distress (53). Indeed, multiple studies have confirmed reciprocal relationships between alcohol consumption and anxiety symptoms (54, 55).

Likewise, our findings regarding stress-induced insomnia align with previous studies indicating that occupational stressors, including excessive workloads, time constraints, and emotionally demanding patient interactions, consistently precipitate sleep disturbances among healthcare professionals (56, 57). Anxiety has emerged as a particularly robust predictor of insomnia severity (58). Recent evidence has revealed that this relationship operates bidirectionally, with reciprocal influences between anxiety and insomnia (59) as well as between stress and sleep quality (60). Furthermore, alcohol use and insomnia have demonstrated important interconnections. Although some individuals adopt drinking as a stress-management strategy, alcohol consumption paradoxically disrupts sleep architecture and continuity, intensifying insomnia severity (61, 62). Overall, these interconnected relationships among perceived stress, anxiety, unhealthy alcohol use, and insomnia create a self-perpetuating cycle that progressively reinforces maladaptive patterns, highlighting the urgent need to have comprehensive intervention strategies that disrupt this detrimental sequence in high-pressure healthcare environments.

### 4.3 The moderating effect of resilience on the relationship between perceive stress/anxiety and unhealthy alcohol use/insomnia among hospital staff (H3)

Regarding H3, resilience significantly moderated the relationship between perceived stress/anxiety and unhealthy alcohol use. This positive association between perceived stress/anxiety and unhealthy alcohol use was stronger among individuals with lower resilience, suggesting that those with limited psychological resources are more susceptible to engaging in stress-induced drinking behaviors. This finding aligns with previous research demonstrating that resilience moderates the indirect pathway from stress through negative emotions to problematic alcohol consumption (63) and that individuals with limited resilience are particularly vulnerable to stress-related drinking (64). Notably, at high resilience levels, the associations between perceived stress/anxiety and unhealthy alcohol use were non-significant, suggesting that high resilience may substantially weaken the association between psychological distress and drinking behavior. Highly resilient individuals likely possess more adaptive coping strategies—such as cognitive reappraisal, problem-solving, and social support-seeking—that reduce their reliance on alcohol as a maladaptive coping mechanism. This interpretation is consistent with the self-medication hypothesis, which posits that individuals use substances to alleviate negative emotional states; high resilience may provide sufficient psychological resources for people to regulate their emotions without resorting to alcohol. In support of this view, resilience has been widely recognized as a protective factor against hazardous drinking (65), and evidence from the COVID-19 pandemic indicates that higher resilience can minimize stress-induced increases in alcohol consumption (66).

Resilience also moderated the relationship between perceived stress/anxiety and insomnia. As resilience levels decreased, the positive association between perceived stress/anxiety and insomnia became progressively stronger. This pattern highlights the protective role of resilience in mitigating sleep disturbances related to psychological distress. Previous studies among nurses and medical students confirm that resilience attenuates stress-induced negative emotions and sleep disturbances (67, 68). Furthermore, strengthening psychological resilience has been link to a reduction in insomnia buffering occupational stress (69). These results indicate that resilience plays a protective role in reducing both substance-related maladaptive coping and sleep disturbances. This evidence highlights the critical need for systematic resilience-building interventions to safeguard the psychological and behavioral health of healthcare workers.

### 4.4 Study limitations and practical implications

This study has limitations that warrant consideration. First, the cross-sectional design precludes causal inferences regarding the relationships among perceived stress, anxiety, resilience, unhealthy alcohol use, and insomnia. The temporal sequence among these variables cannot be determined, and bidirectional relationships remain plausible. Longitudinal studies are needed to clarify the temporal dynamics of these associations. Second, the data were collected from hospital staff at a single tertiary hospital in Hainan Province, China, which may limit the generalizability of findings to healthcare workers in other institutional settings or cultural contexts. Future research should incorporate multi-center and cross-cultural samples to enhance external validity. Third, although the current findings highlight the protective role of resilience, intervention-based research is warranted to determine whether resilience-enhancing training programs can effectively reduce unhealthy alcohol use and insomnia among healthcare workers. Fourth, all of the measures were self-reported, which may introduce social desirability and recall biases, particularly when measuring sensitive outcomes such as alcohol use.

Despite these limitations, the present findings carry considerable practical significance for healthcare institutions. The finding that resilience moderates the associations between perceived stress/anxiety and unhealthy alcohol use/insomnia provides an empirical basis for incorporating resilience-building programs into occupational health frameworks. Hospital administrators may consider integrating evidence-based resilience training, such as cognitive-behavioral stress management, mindfulness-based interventions, and structured peer support programs, into routine staff development activities. In addition, the demographic analyses identified distinct risk profiles among specific subgroups, including staff working extended hours, frontline clinical personnel, male employees, divorced or widowed individuals, and those with lower educational attainment, which can guide the allocation of limited mental health resources toward the populations most in need. Furthermore, the results support the implementation of routine psychological screening protocols that evaluate perceived stress, anxiety, and resilience among hospital staff. Such protocols would facilitate the early identification of at-risk individuals before maladaptive coping patterns become firmly established. By addressing both individual-level psychological resources and institutional-level support structures, healthcare organizations can foster a more protective occupational environment that mitigates the detrimental effects of occupational stress on employee well-being and patient care quality.

## 5 Conclusion

This study examined the relationships among perceived stress, anxiety, unhealthy alcohol use, and insomnia in hospital staff, with particular attention to the moderating role of resilience. The results confirmed that perceived stress and anxiety were positively associated with both unhealthy alcohol use and insomnia, while resilience substantially attenuated these adverse associations. These findings suggest that resilience serves as a critical psychological resource associated with reduced vulnerability to both maladaptive coping behaviors and sleep disturbances among healthcare workers. Despite limitations related to the cross-sectional design and regional sampling, the demographic analyses revealed distinct risk patterns across employee subgroups, highlighting the need for targeted approaches. Future intervention-based studies are warranted to evaluate the effectiveness of resilience-building programs in reducing occupational stress-related health outcomes among healthcare workers.

## Data Availability

The original contributions presented in the study are included in the article/supplementary material, further inquiries can be directed to the corresponding authors.

## References

[1] RotensteinLS RamosMA TorreM SegalJB PelusoMJ GuilleC . Prevalence of depression, depressive symptoms, and suicidal ideation among medical students: a systematic review and meta-analysis. JAMA. (2016) 316:2214–36. doi: 10.1001/jama.2016.1732427923088 PMC5613659

[2] CheungT YipPS. Depression, anxiety and symptoms of stress among Hong Kong nurses: a cross-sectional study. Int J Environ Res Public Health. (2015) 12:11072–100. doi: 10.3390/ijerph12091107226371020 PMC4586662

[3] Marques M Alves E Queirós C Norton P Henriques A. The effect of profession on burnout in hospital staff. Occup Med. (2018) 68:207–10. doi: 10.1093/occmed/kqy039 29546385

[4] ArnetzJE HamblinL EssenmacherL UpfalMJ AgerJ LuborskyM. Understanding patient-to-worker violence in hospitals: a qualitative analysis of documented incident reports. J Adv Nurs. (2015) 71:338–48. doi: 10.1111/jan.1249425091833 PMC5006065

[5] Ishikawa M. Relationships between overwork, burnout and suicidal ideation among resident physicians in hospitals in Japan with medical residency programmes: a nationwide questionnaire-based survey. BMJ Open. (2022) 12:e056283. doi: 10.1136/bmjopen-2021-056283 PMC8915267 35273058

[6] MagnavitaN MeragliaI RiccòM. Anxiety and depression in healthcare workers are associated with work stress and poor work ability. AIMS Public Health. (2024) 11:1223–37. doi: 10.3934/publichealth.202406339802561 PMC11717537

[7] ZhouY GaoW LiH YaoX WangJ ZhaoX. Network analysis of resilience, anxiety and depression in clinical nurses. BMC Psychiatry. (2024) 24:719. doi: 10.1186/s12888-024-06138-839438840 PMC11520162

[8] KimEJ DimsdaleJE. The effect of psychosocial stress on sleep: a review of polysomnographic evidence. Behav Sleep Med. (2007) 5:256–78. doi: 10.1080/1540200070155738317937582 PMC4266573

[9] Åkerstedt T. Psychosocial stress and impaired sleep. Scand J Work Environ Health. (2006) 32:493–501. doi: 10.5271/sjweh.1054 17173205

[10] ZaheedAB ChervinRD SpiraAP ZahodneLB. Mental and physical health pathways linking insomnia symptoms to cognitive performance 14 years later. Sleep. (2023) 46:zsac262. doi: 10.1093/sleep/zsac26236309871 PMC9995792

[11] Sofi F Cesari F Casini A Macchi C Abbate R Gensini GF. Insomnia and risk of cardiovascular disease: a meta-analysis. Eur J Prev Cardiol. (2014) 21:57–64. doi: 10.1177/2047487312460020 22942213

[12] Söderström M Jeding K Ekstedt M Perski A Åkerstedt T. Insufficient sleep predicts clinical burnout. J Occup Health Psychol. (2012) 17:175–83. doi: 10.1037/a0027518 22449013

[13] Philibert I. Sleep loss and performance in residents and nonphysicians: a meta-analytic examination. Sleep. (2005) 28:1392–402. doi: 10.1093/sleep/28.11.1392 16335329

[14] Sadeghniiat-HaghighiK NajafiA EftekhariS TarkhanS. Insomnia and its association with absenteeism: a cross-sectional study among Iranian nursing team. Sleep Sci. (2021) 14:305–10. doi: 10.5935/1984-0063.2020010635087626 PMC8776262

[15] LyuX LiK LiuQ WangX YangZ YangY . Sleep status of psychiatric nurses: a survey from China. Nurs Open. (2022) 9:2720–8. doi: 10.1002/nop2.97234198365 PMC9584482

[16] Tang T Zhang N Qu L Zhang J Yang D Shen S . Mental health and the knowledge and attitude towards insomnia among medical staff in China: a cross-sectional study. BMJ Open. (2026) 16:e109402. doi: 10.1136/bmjopen-2025-109402 PMC12815158 41513414

[17] ZhanY LiuY LiuH LiM ShenY GuiL . Factors associated with insomnia among Chinese front-line nurses fighting against COVID-19 in Wuhan: a cross-sectional survey. J Nurs Manag. (2020) 28:1525–35. doi: 10.1111/jonm.1309432657449 PMC7405094

[18] dos Santos DT Nazário FP Freitas RA Henriques VM de Paiva IS. Alcohol abuse and dependence among Brazilian medical students: association to sociodemographic variables, anxiety and depression. J Subst Use. (2019) 24:285–92. doi: 10.1080/14659891.2018.1562574

[19] ObeidS AkelM HaddadC FaresK SacreH SalamehP . Factors associated with alcohol use disorder: the role of depression, anxiety, stress, alexithymia and work fatigue—a population study in Lebanon. BMC Public Health. (2020) 20:245. doi: 10.1186/s12889-020-8345-132070314 PMC7029557

[20] Medisauskaite A Kamau C. Does occupational distress raise the risk of alcohol use, binge-eating, ill health and sleep problems among medical doctors? A UK cross-sectional study. BMJ Open. (2019) 9:e027362. doi: 10.1136/bmjopen-2018-027362 PMC6530309 31092661

[21] CousinL RoucouxG PetitAS Baumann-CoblentzL TorrenteOR CannafarinaA . Perceived stigma, substance use and self-medication in night-shift healthcare workers: a qualitative study. BMC Health Serv Res. (2022) 22:698. doi: 10.1186/s12913-022-08018-x35610623 PMC9128768

[22] HalsallL IrizarP BurtonS WaringS GilesS GoodwinL . Hazardous, harmful, and dependent alcohol use in healthcare professionals: a systematic review and meta-analysis. Front Public Health. (2023) 11:1304468. doi: 10.3389/fpubh.2023.130446838089041 PMC10715281

[23] Vázquez-Puente EO López-García KS Rafael F. Anxiety and depressive symptoms associated to alcohol consumption in health care workers. Horizon. (2023) 1:e14. doi: 10.56935/hij.v1i3.14

[24] ZhanN XuY PuJ WangW XieZ HuangH. The interaction between mental resilience and insomnia disorder on negative emotions in nurses in Guangdong Province, China. Front Psychiatry. (2024) 15:1396417. doi: 10.3389/fpsyt.2024.139641739176229 PMC11339876

[25] SchwandtML CullinsE RamchandaniVA. The role of resilience in the relationship between stress and alcohol. Neurobiol Stress. (2024) 31:100644. doi: 10.1016/j.ynstr.2024.10064438827175 PMC11140813

[26] WuY SangZQ ZhangXC MargrafJ. The relationship between resilience and mental health in Chinese college students: a longitudinal cross-lagged analysis. Front Psychol. (2020) 11:108. doi: 10.3389/fpsyg.2020.0010832116918 PMC7012791

[27] Cohen S Kamarck T Mermelstein R. A global measure of perceived stress. J Health Soc Behav. (1983) 24:385–96. doi: 10.2307/2136404 6668417

[28] WangZ ChenJ BoydJE ZhangH JiaX QiuJ . Psychometric properties of the Chinese version of the Perceived Stress Scale (PSS-10) in policewomen. PLoS ONE. (2011) 6:e28610. doi: 10.1371/journal.pone.002861022164311 PMC3229602

[29] Spitzer RL Kroenke K Williams JB Löwe B. A brief measure for assessing generalized anxiety disorder: the GAD-7. Arch Intern Med. (2006) 166:1092–7. doi: 10.1001/archinte.166.10.1092 16717171

[30] He X Li C Qian J Cui H Wu W. Reliability and validity of a generalized anxiety disorder scale in general hospital outpatients. Shanghai Arch Psychiatry. (2010) 22:200–3.

[31] Campbell-Sills L Stein MB. Psychometric analysis and refinement of the Connor-Davidson Resilience Scale (CD-RISC): validation of a 10-item measure. J Trauma Stress. (2007) 20:1019–28. doi: 10.1002/jts.20271 18157881

[32] Yu X Zhang J. Factor analysis and psychometric evaluation of the Connor-Davidson Resilience Scale (CD-RISC) with Chinese people. Soc Behav Pers. (2007) 35:19–30. doi: 10.2224/sbp.2007.35.1.19

[33] Saunders JB Aasland OG Babor TF de la Fuente JR Grant M. Development of the Alcohol Use Disorders Identification Test (AUDIT): WHO collaborative project on early detection of persons with harmful alcohol consumption. Addiction. (1993) 88:791–804. doi: 10.1111/j.1360-0443.1993.tb02093.x 8329970

[34] Zhang C Yang GP Li Z Li XN Li Y Hu J . Reliability and validity of the Chinese version on alcohol use disorders identification test. Zhonghua Liu Xing Bing Xue Za Zhi. (2017) 38:1064–7. doi: 10.3760/cma.j.issn.0254-6450.2017.08.013 28847055

[35] Bastien CH Vallières A Morin CM. Validation of the insomnia severity index as an outcome measure for insomnia research. Sleep Med. (2001) 2:297–307. doi: 10.1016/S1389-9457(00)00065-4 11438246

[36] Chung KF Kan KKK Yeung WF. Assessing insomnia in adolescents: comparison of insomnia severity index, athens insomnia scale and sleep quality index. Sleep Med. (2011) 12:463–70. doi: 10.1016/j.sleep.2010.09.019 21493134

[37] Hayes AF. Introduction to Mediation, Moderation, and Conditional Process Analysis: A Regression-Based Approach. New York, NY: Guilford Publications (2017).

[38] Preacher KJ Hayes AF. Asymptotic and resampling strategies for assessing and comparing indirect effects in multiple mediator models. Behav Res Methods. (2008) 40:879–91. doi: 10.3758/BRM.40.3.879 18697684

[39] YiJ KangL LiJ GuJ. A key factor for psychosomatic burden of frontline medical staff: occupational pressure during the COVID-19 pandemic in China. Front Psychiatry. (2021) 11:590101. doi: 10.3389/fpsyt.2020.59010133536948 PMC7848019

[40] Chan SM Au-Yeung TC Wong H Chung RYN Chung GKK. Long working hours, precarious employment and anxiety symptoms among working Chinese population in Hong Kong. Psychiatr Q. (2021) 92:1745–57. doi: 10.1007/s11126-021-09938-3 34373982

[41] UccellaL MascheronaI SeminiS UccellaS. Exploring resilience among hospital workers: a Bayesian approach. Front Public Health. (2024) 12:1403721. doi: 10.3389/fpubh.2024.140372139267645 PMC11390436

[42] WongELY QiuH ChienWT WongCL ChaliseHN HoangHTX . Comparison of resilience among healthcare workers during the COVID-19 pandemics: a multinational cross-sectional survey in southeast Asian jurisdictions. Int J Public Health. (2022) 67:1605505. doi: 10.3389/ijph.2022.160550536618431 PMC9811508

[43] MarzoRR ElSherifM AbdullahMSAMB ThewHZ ChongC SohSY . Demographic and work-related factors associated with burnout, resilience, and quality of life among healthcare workers during the COVID-19 pandemic: a cross-sectional study from Malaysia. Front Public Health. (2022) 10:1021495. doi: 10.3389/fpubh.2022.102149536589987 PMC9800419

[44] Moinuddin A Goel A Saini S Bajpai A Misra R. Alcohol consumption and gender: a critical review. J Psychol Psychother. (2016) 6:1–4. doi: 10.4172/2161-0487.1000267

[45] Pudrovska T Carr D. Psychological adjustment to divorce and widowhood in mid- and later life: do coping strategies and personality protect against psychological distress? Adv Life Course Res. (2008) 13:283–317. doi: 10.1016/S1040-2608(08)00011-7

[46] BeardE BrownJ WestR KanerE MeierP MichieS. Associations between socio-economic factors and alcohol consumption: a population survey of adults in England. PLoS ONE. (2019) 14:e0209442. doi: 10.1371/journal.pone.020944230716098 PMC6361426

[47] Afonse P Fonseca M Pires JF. Impact of working hours on sleep and mental health. Occup Med. (2017) 67:377–82. doi: 10.1093/occmed/kqx054 28575463

[48] ObasiEM BrooksJJ CavanaghL. The relationship between psychological distress, negative cognitions, and expectancies on problem drinking: exploring a growing problem among university students. Behav Modif. (2016) 40:51–69. doi: 10.1177/014544551560179326311191 PMC4740224

[49] Sinha R. How does stress lead to risk of alcohol relapse? Alcohol Res Curr Rev. (2012) 34:432–40. doi: 10.35946/arcr.v34.4.07 PMC3788822 23584109

[50] SebenaR El AnsariW StockC OrosovaO MikolajczykRT. Are perceived stress, depressive symptoms and religiosity associated with alcohol consumption? Subst Abuse Treat Prev Policy. (2012) 7:21. doi: 10.1186/1747-597X-7-2122640549 PMC3395565

[51] Waithera HW Ndumwa HP Njiro BJ Chande-Mallya R Julius W Swahn M . Alcohol use disorders among healthcare professionals: a call for action. Health Promot Int. (2024) 39:daae121. doi: 10.1093/heapro/daae121 39397748

[52] Kolonne T Mudalige K Dissanayaka G Rathnayake K Jayathilaka R Rajamanthri L . Investigating the associations between alcohol consumption and prevalence of anxiety using multiple correspondence analysis. Int J Ment Health Addict. (2025) 1–16. doi: 10.1007/s11469-025-01561-8

[53] Onaemo VN Chireh B. Alcohol, depression, and anxiety. In: Handbook of the Behavior and Psychology of Disease. Springer: Cham (2025) 2205–25. doi: 10.1007/978-3-031-73363-5_130

[54] DyerML HeronJ HickmanM MunafòMR. Alcohol use in late adolescence and early adulthood: the role of generalized anxiety disorder and drinking to cope motives. Drug Alcohol Depend. (2019) 204:107480. doi: 10.1016/j.drugalcdep.2019.04.04431706711 PMC6891250

[55] SenarathneB PalliyaguruD OshiniA GamageJ JayathilakaR RajamanthriL . Evaluating the synergy: anxiety prevalence and alcohol consumption patterns in high-income countries using Granger causality analysis. BMC Public Health. (2025) 25:220. doi: 10.1186/s12889-025-21402-639828695 PMC11744946

[56] Mosadeghrad AM. Occupational stress and its consequences: implications for health policy and management. Leadersh Health Serv. (2014) 27:224–39. doi: 10.1108/LHS-07-2013-0032

[57] Yeh YC Lin BYJ Lin WH Wan TT. Job stress: its relationship to hospital pharmacists' insomnia and work outcomes. Int J Behav Med. (2010) 17:143–53. doi: 10.1007/s12529-009-9066-0 19924543

[58] Ohayon MM Roth T. Place of chronic insomnia in the course of depressive and anxiety disorders. J Psychiatr Res. (2003) 37:9–15. doi: 10.1016/S0022-3956(02)00052-3 12482465

[59] MaoT GuoB RaoH. Unraveling the complex interplay between insomnia, anxiety, and brain networks. Sleep. (2024) 47:zsad330. doi: 10.1093/sleep/zsad33038195150 PMC10925950

[60] PetakA MaričićJ. The role of rumination and worry in the bidirectional relationship between stress and sleep quality in students. Int J Environ Res Public Health. (2025) 22:1001. doi: 10.3390/ijerph2207100140724068 PMC12294785

[61] Brower KJ. Assessment and treatment of insomnia in adult patients with alcohol use disorders. Alcohol. (2015) 49:417–27. doi: 10.1016/j.alcohol.2014.12.003 25957855

[62] RoehrsTA RothT. Sleep disturbance in substance use disorders. Psychiatr Clin North Am. (2015) 38:793–804. doi: 10.1016/j.psc.2015.07.00826600109 PMC4660250

[63] WangY ChenX. Stress and alcohol use in rural Chinese residents: a moderated mediation model examining the roles of resilience and negative emotions. Drug Alcohol Depend. (2015) 155:76–82. doi: 10.1016/j.drugalcdep.2015.08.01426342628 PMC4586155

[64] Wong MCS Huang J Wang HHX Yuan J Xu W Zheng ZJ . Resilience level and its association with maladaptive coping behaviours in the COVID-19 pandemic: a global survey of the general populations. Glob Health. (2023) 19:1. doi: 10.1186/s12992-022-00903-8 PMC9808687 36597129

[65] CusackSE WrightAW AmstadterAB. Resilience and alcohol use in adulthood in the United States: a scoping review. Prev Med. (2023) 168:107442. doi: 10.1016/j.ypmed.2023.10744236736834 PMC9974891

[66] Tudehope L Lee P Wiseman N Dwirahmadi F Sofija E. The effect of resilience on the relationship between perceived stress and change in alcohol consumption during the COVID-19 pandemic in Queensland, Australia. J Health Psychol. (2022) 27:2696–713. doi: 10.1177/13591053211062351 34886691

[67] TempskiP SantosIS MayerFB EnnsSC PerottaB ParoHBMS . Relationship among medical student resilience, educational environment and quality of life. PLoS ONE. (2015) 10:e0131535. doi: 10.1371/journal.pone.013153526121357 PMC4486187

[68] ChengZ TaoY LiuT HeS ChenY SunL . Psychology, stress, insomnia, and resilience of medical staff in China during the COVID-19 policy opening: a cross-sectional survey. Front Public Health. (2023) 11:1249255. doi: 10.3389/fpubh.2023.124925537693701 PMC10485264

[69] Cao Q Wu H Tang X Zhang Q Zhang Y. Effect of occupational stress and resilience on insomnia among nurses during COVID-19 in China: a structural equation modelling analysis. BMJ Open. (2024) 14:e080058. doi: 10.1136/bmjopen-2023-080058 PMC11227768 38969387

